# Optical Genomic Mapping and Next-Generation Sequencing Identified Retrotransposon Insertion and Missense Variant Disrupting *PARN* Gene in Dyskeratosis Congenita

**DOI:** 10.1155/humu/9290736

**Published:** 2025-08-22

**Authors:** Qiaoyu Cao, Anqi Zhao, Zhoukai Long, Xinyi Wang, Chaolan Pan, Yumeng Wang, Wei He, Haisheng Huang, Fuying Chen, Chenfei Wang, Xiaoxiao Wang, Luming Sun, Jingjun Zhao, Ming Li

**Affiliations:** ^1^Department of Dermatology, Children's Hospital of Fudan University, National Children's Medical Center, Shanghai, China; ^2^Institute of Dermatology, Shanghai Jiao Tong University School of Medicine, Shanghai, China; ^3^Shanghai We Health Biomedical Technology Co. Ltd., Shanghai, China; ^4^Department of Fetal Medicine, Shanghai First Maternity and Infant Hospital, Tongji University, Shanghai, China

**Keywords:** dyskeratosis congenita, optical genome mapping, *PARN* gene, retrotransposons, structural variant

## Abstract

Dyskeratosis congenita (DC) is an inherited bone marrow failure syndrome characterized by defects in telomere biology and clinical manifestations such as nail dystrophy, skin pigmentation abnormalities, and mucosal leukoplakia. Here, using whole exome sequencing (WES), whole genome sequencing (WGS), optical mapping sequencing (OGM), third-generation sequencing, and mRNA sequencing, we diagnosed a participant with *PARN* gene complex compound heterozygous variants. In addition, protein structure simulation, immunohistochemistry, and western blot were conducted to investigate the structure and expression level of the PARN protein. WES revealed a maternal *PARN* variant, c.204G>T (p.Gln68His) (NM_002582.3). An insertion variant in the *PARN* gene from the father was identified by OGM and mRNA sequencing. Third-generation sequencing results determined the insertion position of the SINE-VNTR-Alu (SVA) transposon and its size (2537 bp), which was found to lead to a premature stop codon (p.Gly469delinsGlu∗). The PARN protein level of the parents was reduced due to complex heterozygous variants. Overall, OGM diagnosed the structural variants of the participant with DC, supplementing the disease variant spectrum of DC. This case highlights a novel disease-causing structural variant and the importance of transposon analysis in a clinical diagnostic setting.

## 1. Introduction

Dyskeratosis congenita (DC) is a progressive, inherited bone marrow failure syndrome caused by a telomere biology disorder (TBD) [1]. Clinical features include reticular pigmentation of the skin, nail dystrophy, and oral leukoplakia, which may be accompanied by serious complications including bone marrow failure, immune deficiency, malignancy, and pulmonary fibrosis [2]. Previously reported pathogenic genes include poly(A)-specific ribonuclease (*PARN*), *DKC1*, *TERT*, *TERC*, *TINF2*, *C16orf57*, *NOLA2*, *NOLA3*, *RTEL1*, and *WRAP53/TCAB1* [3]. DC inheritance pattern includes autosomal recessive inheritance, autosomal dominant inheritance, and X-linked inheritance. DC, a TBD, is characterized by deficiencies in the synthesis and preservation of telomeric DNA. This critical process is mediated by telomerase, a telomere-specific reverse transcriptase that utilizes a segment of its RNA subunit as a template to maintain the integrity of chromosome terminal regions [4, 5]. PARN is a key enzyme involved in controlling mRNA stability, ribosome biogenesis, maintenance of telomeres, and the DNA damage response [6].

Whole exome sequencing (WES) is widely used for genetic testing of hereditary dermatosis in our hospital; however, it occasionally fails to identify pathogenic variants, particularly in recessive disease. Copy number variants (CNVs) or structural variants (SVs) have sometimes been identified as the underlying cause. So far, no cases have been reported where SV of *PARN* cause DC.

In the study, we describe a previously unreported SV (chr16:14,553,337–14,553,338 insertion) and a recurrent variant of the *PARN* gene (c.204G>T, p.Q68H) detected in an 11-year-old Chinese boy with DC.

## 2. Materials and Methods

Participant, immunohistochemistry of skin biopsy specimens [7], DNA isolation, genetic analysis, DNA labeling, data collection, genome assembly, variant calling, data visualization and Sanger sequencing, RNA sequencing (RNA-Seq), molecular dynamics simulation, structure analysis, and immunoblot analysis are described in Supporting Information 1: Supporting Methods.

## 3. Results

### 3.1. Dermatological Examination

**Figure 1:**
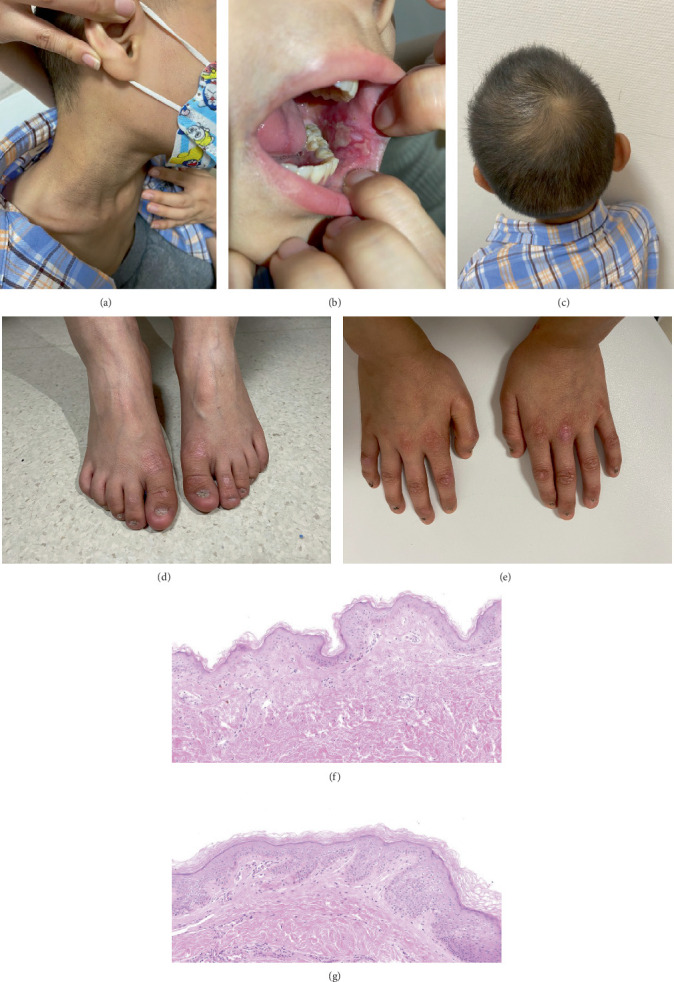
Clinical characteristics of the proband. (a) Reticulated hyperpigmentation over the neck. (b) Leukoplakia. (c) Microcephaly. (d, e) Dystrophic nails. (f, g) Hematoxylin and eosin stain of (f) patient and (g) normal skin in the underarm.

The proband is an 11-year-old Chinese boy with specific dermatological features. He exhibits pigmentation variegation on the neck and underarms, along with the disappearance of skin lines, nail atrophy, longitudinal ridge torsion, and gradual reduction, even absence, of nails. Additionally, white patches and thickening of buccal mucosa appeared on both the buccal mucosa and the back of the tongue. The participant also displays microcephaly, progeroid facial features, and premature alopecia (Figures 1a, 1b, 1c, 1d, and 1e). His commissures were dry and irritated, and he experienced difficulty when opening his mouth wide. Complete blood counts showed RBC 2.87 × 10^9^/L (normal range 4.2 ~ 5.7 × 10^9^/L), WBC 6.34 × 10^9^/L (normal range 4.3 ~ 11.3 × 10^9^/L), hemoglobin 93 g/L (normal range 118~156 g/L) with MCV 104.9 fl, and platelet count of 44 × 10^9^/L (normal range 167 ~ 453 × 10^9^/L) in 2023, which revealed mild thrombocytopenia. Telomere length analysis indicates that the average telomere length of the proband is 7.09 kb, which is shorter than the average length of people in this age group (Supporting Information 6: Figure S4). The proband's mother is 36 years old, and his father is 39 years old. Clinical evaluation of both parents revealed no abnormalities in dermatological assessments, growth parameters, or developmental milestones. Neither parent has a medical history of bone marrow failure, immunodeficiency, or congenital anomalies.

### 3.2. Histopathological Findings

Skin biopsy from his underarm skin with pigmentation plantar demonstrated that the participant's skin epidermis is obviously thinner and lacks epidermal protrusions compared to normal skin (Figure 1f,g).

### 3.3. Genetic Characteristics

**Figure 2:**
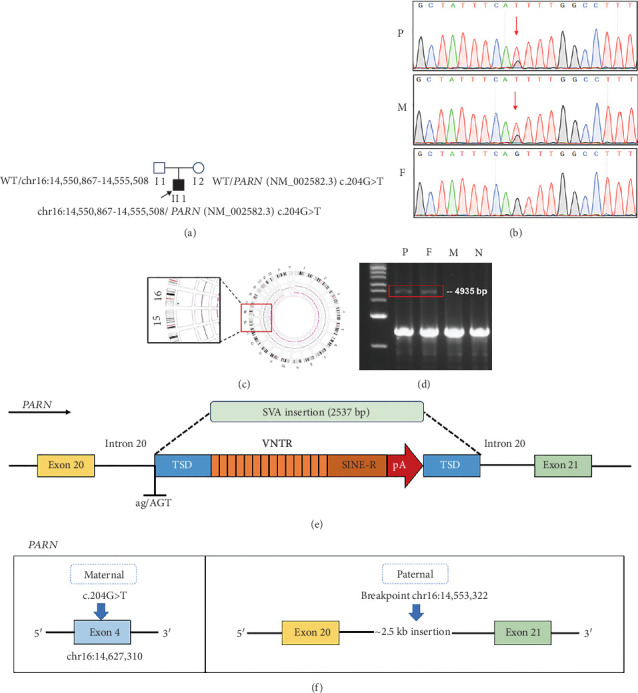
Genetic analysis of the patient at the DNA level. (a) Family pedigree of the proband (arrows) with heterozygous *PARN* pathogenic variants described in this article. (b) A heterozygous *PARN* variant (NM_002582.3, c.204G>T) identified by exome sequencing. Sanger sequencing of genomic DNA confirmed the variant and its maternal origin (P, proband; M, mother; F, father). (c) Circos of optical genomic mapping. Structural variation of the proband was indicated by red boxes in the circos figure. The blue dots represent insertions. (d) Gelatinization of long fragment amplification products from third-generation sequencing. The patient and his father had a mutant band of 4935 bp (N, normal). (e) Diagram of SVA retrotransposon insertion into the PARN gene. The inserted SVA length is 2537 bp. The structure is VNTR, sin-r, and poly(A) tails, flanked by TSD. The sequence ag/AGT is retained by introns. (f) An overview of the patient's *PARN* variants. The missense variant is Exon 4 of *PARN* (NM_002582.3, c.204G>T), and the structural variant insertion is in Intron 20 of *PARN*, of which one of the breakpoints is chr16:14,553,322.

The WES identified a missense variant of the PARN gene (c.204G>T, p.Gln68His), confirmed by Sanger sequencing to be maternally inherited (Figure 2b). This variant is extremely rare, with an allele frequency of 0.000004 in the gnomAD database, and has been previously reported in association with TBD in two independent studies [8, 9], further supporting its pathogenic relevance.

Using OGM, we discovered another structural variation in the *PARN* gene, visualized in the circus figure (Figure 2c). The distance between the labels of *PARN* gene chr16:14,550,867–14,555,508 (Ref, GRCh38/hg18 reference sequence) was 7160 bp. In contrast, the normal reference genome was 4641 bp between labels chr16:14,550,867–14,555,508, indicating an insertion variant in the *PARN* gene of the participant with an insertion fragment length of approximately 2520 bp. Long-range PCR with specific primers (Primer 1, Supporting Information 7: Table S1) detected genome-level transposons inserted into Intron 20 in the proband and father, confirming paternal inheritance, and the length of the amplified product was about 4935 bp (Figure 2d).

The predicted breakpoint position is chr16:14,553,322, and the inserted sequence is SINE-VNTR-Alu (SVA) transposon (Primer 2, Supporting Information 7: Table S1). The amplification process was stable and showed good specificity across three generations of sequencing, using the original primers for joining the joint series (Primer 3, Supporting Information 7: Table S1). The mutant amplification products (4935 bp) were gel-isolated and sequenced. Comparison with the original connector sequence and primers finally obtained the specific sequences of the SVA transposons inserted in the *PARN* gene (Supporting Information 4: Figure S2). The specific sequence of SVA transposons inserted in the PARN gene is showed in Supporting Information 2: Supporting Sequence. Based on the third-generation sequencing results, the insertion position of the SVA transposon was determined to be chr16:14,553,337–14,553,338, and the insertion sequence size was 2537 bp. In summary, the missense variant is located in Exon 4 of *PARN* (NM_002582.3, c.204G>T), and the SV insertion is in Intron 20 of *PARN*, with one of the breakpoints being chr16:14,553,322 (Figure 2a,f).

Splicing analysis by mRNA sequencing indicated that part of Intron 20 was retained between Exons 20 and 21 of the *PARN* gene in the child and the father, but no significant intron retention was found in the mother (Supporting Information 3: Figure S1a). Specific primers were designed at Intron 20 (Primer 4, Supporting Information 7: Table S1). There was an insertion of approximately 828 bp in Intron 20 from the SVA retrotransposon (RE) sequence, of which the wild-type band was 1920 bp and the mutant band was 2748 bp (Supporting Information 3: Figure S1b). A presumed stop codon appeared immediately after codon 468 of *PARN* (*PARN* p.Gly469delinsGlu∗) (Supporting Information 3: Figure S1c,d). Structural analyses and molecular dynamics simulations were conducted on the wild-type protein and the Gln68His variant protein (Supporting Information 3: Figure S1e, Supporting Information 5: Figure S3).

### 3.4. Immunohistochemical and Western Blot

Immunohistochemical and western blot results indicated that the expression level of PARN protein in the epidermal tissue of the patient was lower than that of the normal epidermis (Supporting Information 3: Figure S1f,g), indicating that the point variant and transposon insertion led to abnormal expression of the PARN protein. Furthermore, immunohistochemistry indicated that PARN was concentrated in the nucleoli of the children, with an expression level lower than that of normal individuals.

## 4. Discussion

DC, alternatively termed Zinsser–Engman–Cole syndrome, is a rare progressive syndrome resulting in bone marrow failure [10]. It is identified by a triad of symptoms: reticular skin pigmentation, oral leukoplakia, and nail dystrophy [11]. The disease's primary causes are telomerase dysfunction. Fatality often results from bone marrow failure, infection, fatal lung complications, or malignancy, leading to premature death [12].

The PARN protein comprises six domains: N-terminal nuclease domains, 3-histidine containing domain, a conserved arginine, a nuclear localization signal, an RNA recognition motif, and a C-terminal domain [13, 14]. Biallelic *PARN* variants are associated with overlapping of DC and Hoyeraal–Hreidarsson syndrome (HHS) [15]. While DC presents with classic mucocutaneous features, HHS manifests additional severe phenotypes, including bone marrow failure, immunodeficiency, intrauterine growth retardation, developmental delay, and cerebellar hypoplasia [16]. To date, biallelic *PARN* variants have been reported in only 12 patients with DC/HHS [8, 9, 15–18]. Among 93 distinct *PARN* variants identified in TBDs, 41 (44.1%) were classified as pathogenic/likely pathogenic, showing no obvious mutational hotspots [19]. Notably, *Parn* knockout mice exhibit early embryonic lethality, indicating that the *PARN* mutations identified in patients are either hypomorphic or accompanied by compensatory mechanisms, for instance, the upregulation of alternative deadenylases [9]. In our study, we identified a previously unreported variant (chr16:14,553,337–14,553,338 insertion, p.Gly469delinsGlu∗) and a deleterious variant (c.204G>T, p.Q68H) in the *PARN* gene detected in an 11-year-old Chinese boy presenting with clinical features consistent with TBD. The biallelic variant in *PARN* disrupts its deadenylase activity, leading to reduced telomerase activity, supported by the patient's extremely short telomeres, a hallmark of TBDs [8]. Homozygous c.204G>T in *PARN* has been shown to cause telomere shortening in a patient with HHS, aligning with our observations [9].

Typically, genetic counseling faces difficulties with SVs due to the lack of routine molecular breakpoint characterization. Notably, optical genome mapping can offer a higher resolution than traditional methods, allowing for the detection of all classes of SVs [20]. Consequently, it emerges as a crucial tool in genomic technology for identifying SVs in patients with monogenic disorders, including insertions.

Utilizing OGM, we confirmed the insertion located between g.14,550,867 and g.14,555,508 of Chromosome 16 (hg38). The predicted breakpoint position is at chr16:14,553,322 (hg38), with the insertion sequence identified as the SVA transposon. RNA-level analysis revealed an approximately 828 bp insertion in Intron 20 from the RE sequence. Despite initial difficulties in obtaining the specific sequence, we succeeded through repeated trials and continuous design of new primers, achieving a clear insertion of the 2560 bp RE sequence via targeted third-generation sequencing. This marks the first reported case of DC with a partial insertion of *PARN* combined with a heterozygous *PARN* gene missense variant. Meanwhile, the levels of PARN protein of our participant indicated that this complex heterozygous variant could be harmful to the normal function of the gene.

REs are mobile genetic elements that propagate via RNA intermediates, inserting into host genomes through retrotransposition. Classified into long terminal repeats (LTRs) and non-LTR subtypes, the latter include short interspersed nuclear elements (SINEs) (e.g., Alu elements) and long interspersed nuclear elements (LINEs) (e.g., LINE-1, L1). Alu elements (∼300 bp), the most abundant REs in humans, rely on L1-encoded enzymes for retrotransposition despite lacking coding capacity [21]. SVAs, the youngest non-LTR family (∼2700 copies in the reference genome), are complex REs capable of disrupting gene function [22]. Insertions into critical genomic regions—such as exon–intron junctions—can induce pathogenic splicing alterations, as observed in our proband with DC. Here, SVA-mediated retention of *PARN* Intron 20 led to reduced protein expression, linking RE activity to disease etiology.

## 5. Conclusions

In our clinical report, we identified complex compound heterozygous pathogenic *PARN* variants using WES, OGM, and the third-generation sequencing. Breakpoints were confirmed by long-range PCR and Sanger sequencing, both clinically and genetically. This case represents a milestone in the application of OGM, being the first to diagnose an undetermined heterozygous case within PARN-DC. It is also the inaugural report of patients exhibiting typical characteristics of DC and an insertion variant of the *PARN* gene. This case underscores the significance of structural genome variants in monogenic diseases, the role of REs in pathogenicity, and the clinical utility of OGM in uncovering SVs. The integration of these techniques in cases linked to SVs in the *PARN* gene aids in understanding the molecular basis of DC. Potentially, we revealed the abnormal variants of the *PARN* gene and expanded the spectrum of DC, which has guiding significance for the diagnosis.

## Data Availability

All data described in this manuscript is available in the main text or supporting files.

## Supporting Information

Supporting Information 1 Supporting Methods: Detailed procedures for telomere assays, immunohistochemistry, optical genome mapping, RNA sequencing, and molecular dynamics simulation.

Supporting Information 2 Supporting Sequence: The complete inserted sequence of the SVA retrotransposon validated in this case.

Supporting Information 3 Figure S1: Genetic analysis at the RNA and protein levels, including intron retention, aberrant transcripts, protein structural changes, and reduced PARN expression.

Supporting Information 4 Figure S2: The full inserted sequence of the SVA retrotransposon within the PARN gene, including annotated splicing and TSD regions.

Supporting Information 5 Figure S3: Molecular dynamics simulation results comparing the stability of wild-type and mutant PARN proteins.

Supporting Information 6 Figure S4: Telomere length analysis, showing reduced telomere length in the proband compared to age-matched controls.

Supporting Information 7 Table S1: Lists of all primers used in long-range PCR and RNA validation experiments.
