# Supplementary material for: Optical Genomic Mapping and Next-Generation Sequencing Identified Retrotransposon Insertion and Missense Variant Disrupting PARN Gene in Dyskeratosis Congenita
Source: Hum Mutat. 2025 Aug 22;2025:9290736. doi: 10.1155/humu/9290736 (PMC12396913; doi:10.1155/humu/9290736)
Supplement: Supporting Information 1 — Supporting Methods: Detailed procedures for telomere assays, immunohistochemistry, optical genome mapping, RNA sequencing, and molecular dynamics simulation. [file 9290736.f1.docx]

**SUPPLEMENTARY METHODS**

**Participant**

This study has secured written informed consent from all participants, received approval from the Ethics Review Committee of Fudan University Children's Hospital, and adheres to the Declaration of Helsinki. Collecte serum samples from both affected and unaffected individuals. A 4mm³ skin biopsy was conducted on the underarm skin of the proband, with normal skin samples obtained during the pigmented nevus removal surgery.

**Telomere Restriction Fragment Assay**

Telomere Restriction Fragment Assay (TRF) quantitative analysis was employed to quantify telomere lengths in approximately 30 million telomeres across 300,000 cells per sample. Specifically, genomic DNA was first digested with restriction enzymes HinfI and RsaI, followed by separation of telomeric fragments through pulsed-field gel electrophoresis. Southern blot technology was then utilized to detect telomeric repeat sequences, and density scanning was performed to calculate telomere length distributions and average values.

**Immunohistochemistry of skin biopsy specimens**

Skin samples embedded in paraffin were subjected to pathological analysis using hematoxylin-eosin (HE) and immunohistochemistry (IHC) staining, following established protocols^[7]^. PARN monoclonal antibody (Abcam, ab188333; 1:100 dilution) was used as the primary antibody.

**DNA isolation and genetic analysis**

Genomic DNA was extracted, followed by whole-exome sequencing and Sanger sequencing. The cDNA reference sequences were compared with previously reported sequences and screened in the OMIM, ClinVar gnomAD, and HGMD databases. Functional effects were predicted using SIFT and Polyphen2, with conservative analysis of the mutated amino acid sequences.

**DNA labeling, data collection, genome assembly, variant calling, data visualization and Sanger sequencing**

To label DNA, DNA labeling kits from Bionano Genomics (San Diego, CA, USA) were employed to label ultra-high molecular weight (UHMW) DNA molecules. Subsequently, 750 ng of genomic DNA (gDNA) was labeled with DL-green fluorophore and the direct labeling enzyme (dl-1) to eliminate excess fluorophore and reverse stain the DNA backbone overnight. The Saphyr instrument can allow data quantification and visualization.

For data collection, electrophoresis was conducted on fluorescently labeled DNA, which was then loaded into the nanochannel array of a Saphyr Chip (Bionano Genomics). Subsequently, the Bionano instrument was used for automated imaging of the linearized DNA. DNA molecules were analyzed, generating 600 Gb of data per sample over 12–24 hours. The DNA backbone (stained with DL-green) and fluorescent labels on each molecule were detected using Bionano image detection software, available on the supported website. Raw files from the Bionano Saphyr mapping platform in BNX format underwent filtering based on a minimum length of 150 kb and 9 labeled sites per single molecule. Areas with low individual assembly coverage (<92×, or 46× for chr Y) were classified as having low coverage.

For genome assembly and variant calling, we established a unique single-molecule map by defining a series of label locations on a single DNA molecule. To create the reference map, we conducted in silico DLE1 digestion of the human reference genome (GRCh38, hg38), employing predetermined software parameters for comparison. Single molecules were assembled *de novo* into consensus genome maps using Bionano Solve v3.5.1. The analysis of the Bionano data involved the utilization: an SV pipeline for detecting of small SVs and comparing consensus genome maps with reference genome maps and a CNV pipeline for recovering large unbalanced aberrations based on normalized coverage. Filtering out potential artifacts, common SV events, and highly repetitive regions of the genome like segmental duplication, we activated the hg38 DLE-1 SV mask.

We optimized the filter settings using data, following a prior analysis of OGM results to reduce the number of variants. The BED SV and CNV overlap precisions were set at 12 kb and 500 kb, respectively. We applied the following confidence values: CNV = 0.99, inversion = 0.7, inter-translocation =0.65, intra-translocation = 0.3, insertion/deletion = 0, and duplications = -1. Only segments > 500 kb were considered for CNV calls. The minimal breakpoint region was defined by the closest DLE mark location boundary to the crossover points on each chromosome. We retained SVs in other population control samples.

For visualization, the Bionano Access software was used for genome mapping and manual result examination. Circos plots were constructed using the software from https://bionanogenomics.com/support-page/ bionano-access/. While some cases exhibited novel SVs with breakpoints in centromeres, constitutive heterochromatin stretches, or the p arm of acrocentric chromosomes detailed investigations into these regions were beyond the scope of this study due to a lack of reference maps in those areas.

**RNA sequencing (RNA-seq)**

The cDNA Library was prepared by TruSeq strand mRNA Library Prep Kit (Illumina). Sequencing was performed on the Illumina NovaSeq 6000 System (Illumina). Perform data quality control, read filtering and basic calibration. With STAR software, the filtered data was compared with the reference human genome (GRCh37/hg19). Gene level quantification was performed by RSEM (v1.2.28). Use Integrative Genomics Viewer to visualize the data.

**Molecular dynamics simulation and structure analysis**

Protein sequences were downloaded from the NCBI protein database for wild-type and mutant protein construction by Alphafold algorithm. Molecular dynamics simulation was conducted with Gromacs v5.14, employing the Gromos53a6 molecular force field and the SPC water model, over a duration of 50 ns. Initially, the protein structure was placed in a cubic periodic box, with periodic boundary conditions applied in the three dimensions, maintaining a minimum distance of 1.0 nm between the box boundary and protein. Add counterions to neutralize the charge of the protein system. Use the Particle-Mesh-Ewald method to calculate the electrostatic interaction, and the motion of each atom was calculated by the leapfrog algorithm. Energy minimization was carried out with 400 steps using the steepest descent energy method. Each simulation system was subjected to a 50 ps position constraint. The initial velocity for the molecular dynamics simulations was randomly set. Data result graphs were generated using PyMOL, VMD, and Origin v8.5 software. The root mean square deviation (RMSD) of the protein was calculated with the gmx and rms tools, and the structure diagram and curve graph were created with PyMOL and Origin v8.5 software, respectively.

**Immunoblot analysis**

Cell protein lysates were extracted and subjected to SDS-PAGE electrophoresis, followed by membrane transfer. Human PARN primary antibody (Abcam, ab188333; 1:1000 dilution) and horseradish peroxidase-conjugated goat anti-rabbit secondary antibody (Bio-Rad, 170-5046; 1:15,000 dilution) were used for antibody selection. Chemiluminescence detection was carried out using the Clarity Western ECL substrate (Bio-Rad) after washing. Actin (Santa Cruz Biotechnology, sc-1615; 1:1000 dilution) was chosen as the reference antibody. Chemiluminescence signals were captured using the Bio-Rad ChemiDoc Touch imaging system. Image J software was employed for image processing and statistical analysis.
