# Supplementary material for: Optical Genomic Mapping and Next-Generation Sequencing Identified Retrotransposon Insertion and Missense Variant Disrupting PARN Gene in Dyskeratosis Congenita
Source: Hum Mutat. 2025 Aug 22;2025:9290736. doi: 10.1155/humu/9290736 (PMC12396913; doi:10.1155/humu/9290736)
Supplement: Supporting Information 2 — Supporting Sequence: The complete inserted sequence of the SVA retrotransposon validated in this case. [file 9290736.f2.docx]

**SUPPLEMENTARY SEQUENCE**

**The specific sequence of SVA transposons inserted in the PARN gene.**

(The green filled area is intron splicing site, the yellow filled area is TSD, and the red letter area is CDS of SVA)

>gDNA

...ccatggcagaatcccaccaatcctaggcatacttttaggagttgtttggtaatcatgaggaattcataacagttctccaaattagctcagacttagtttcatcaaagagtcactggtttacttgatcacttttcacccatcaatatttcatcataaatttgtcttgtagTGCAGCCTAAACGTGATCATGTTCTCCATGTGACATTCCCCAAAGAATGGAAAACCAGCGACCTTTACCAGCTTTTCAGTGCCTTTGgtaagtcgcaagttcagatgtactttttagggttttgctattcatttggtataggtggtcagaagtagaaagatagttttggcctgcgtataaaagaatgttaatattcaaggttttaatttcactctttgaattttctggtttaggatactgttatctttgtgctaatatcatctgagaaacttttaatagcacctcacaatacagagctattaattcatggaaagcaaaacaccgtgatgaggactgtccttactactgaccagggaaaataagctgtggctaagcataaataatttttaagattaaactcacagggtttgtgtgtttcataatggcaggggaggtcttctctgctttagattaatcaaagtgacagatgtttgggtagatcaggatttgtgatacgtgtatgaggtagggcagtccttctctttccttttttaccaggtcatgtgattaggttcgtttgagcagtctcctgataagtgtttatgaagaccattctgattgtaaacccacagcaatgcctcttattttaacaaacgaagcatttaagctattctctttggaaattcttggataaagtgaaggccacaaaacctaaatgtccagtagtaagaaagtttagtaaatgtttatgtatcttctagtatgtagccatttaaaaaatatttgaggagtttattaaaagaagttaaagtgcttgtattttaacgtttagAGTAAAAAGCAGATACGAGATGTGGAGCCGAAGCTGGACTGTACTGCTGCCATCTCGGCTCACTGCAACCTCCCCGCCTGATTCTCCTGCCTCAGCCTGCCAAGTGCCTGCGATTGCAGGCACGCGCCACCACGCCTGACTGGTTTTGGTGGAGACGGGGTTTCGCTGTGTTGGCCGGGCCGGTCTCCAGCCCCTAACCGCGAGTGATCCGCCAGCCTTGGCCTCCCGAGGTGCCGGGATTGCAGACGGAGTCTCGTTCACTCAGTGCTCAATGGTGCCCAGGCTGGAGTGCAGTGGCGTGATCTCGGCTCACTACAACCTACACCTCCCAGCCGCCTGCCTTGGCCTCCCAAAGTGCCGAGATTGCAGCCTCTGCCCGGCCGCCACCCCGTCTGGGAAGTGAGGAGTGTCTCTGCCTGGCCGCCCATCGTCTGGGATGTGAGGAGCCCCTCTGCCTGGCTGCCCAGTCTGGAAAGTGAGGAGCGTCTCCGCCCGGCCGCCATCCCATCTAGGAAGTGAGGAGCGCCTCTTCCCAGCCGCCATCACATCTAGGAAGTGAGGAGCGTCTCTGCCCGGCCGCCCATCGTCTGAGATGTGGGGAGCGCCTCTGCCCCACCGCCCCATCTGGGATGTGAGGAGCGCCTCTGCCCGGCCGAGACCCCGTCTGGGAGGTGAGGAGCGTCTCTGCCCGGCCGCCCCGTCTGAGAAGTGAGGAGACCCTCTGCCTGGCAACCACCCCGTCTGAGAAGTGAGGAGCCCCTCCGCCCGGCAGCCGCCCCGTCTGAGAAGTGAGGAGCCTCTCCGCCCGGCAGCCACCCCATCTGGGAAGTGAGGAGCGTCTCCACCCAGCAGCCACCCCGTCCGGGAGGGAGGTGGGGGGGGTCAGCCCCCCGCCCGGCCAGCCGCCCCATCCGGGAGGGAGGTGGGGGGTCAGCCCCCCCGCCCGGCCAGCCGTGCCATCCGGGAGGGAGGTGGGGGGGGTCAGCCCCCCGCCTGGCCAGCCGTGCCGTCCGGGAGGGAGGTGGGGGGGGTCAGCCCCCTGCCCGGCCAGCCGCCCCGTCCAGGAGGTGAGGGGCGCCTCTGCCCGCCCGCCCCTACTGGGAAGTGAGGAGCCCCTCAGCCCGGCCAGCCACCCCGTCTGGGAGGGATATGGGGGGGTCAGCCCCCCCCACCCGGCCAGCCGCCCCGTCCAGGAGGGAGGTGGGGGGGTCAGCCCCCCGCCCGGCCAGCCGCCCCGTCCGGGAGGGAGGTGGGGGGGGTCAGCCCTCCGCCCGGCCAGCCGCCCCGTCTGGGAGGTGAGGGGCGCCTCTGCCCGGCCGCCCCTACTGGAAAGTGAGGAGCCCCTCTGCCCGGCCAGCCGCCCCGTCCGGGAGGGAGGTGGGGGGGTCAGCCCCCCGCCCGGCCAGCCGCCCTGTCCGGGAGGGAGGTGGGGGGGGTCAGCCCTCCGCCCGGCCAGCTGCCCCGTCCGGGAGGGAGGTGGGGGGGTCAGCCCCCCGCCCGGCCAGACGCCCCGTCCGGGAGGGAGGTGGGGGGGGTCAGCCCCCCTGCCCGGCCAGCCGCCCCGTCCGGGAGGTGAGGGGCGCCTCTGCCCGGCCGCCCCTACTGGGAAGTGAGGAGCCCCTCTGCCCAGCCAGCCGCCCCGTCCGGGAGGGAGGTGGGGGGGTCAGCCCCCCGCCCGGCCAGCCGCCCCGTCCGGGAAGGAGGTGGGGGGGGTCAGCCCCCCCCGCCCGGCCAGCCGCCCCGTCCGGGAGGTGAGGGGCGCCTCTGCCCGGCCGCCCCCTACTGGGAAGTGAGGAGCCCCTCTGCCTGGCCAGCCGCCCCGTCCGGGAGGGAGGTGGGGGGGTCAGCCCCCTGCCCGGCCAGCCGCCCCGTCCGGGAGGTGAGGGGCGCCTCTGCCCGGCCGCCCCTACTGGGAAGTGAGGAGCCCCTCTGCCCGGCCACCACCCCGTCTGGGAGGTGTGCCCAGCAGCTCATTGAGAACGGGCCAGGATGACAATGGCGGCTTTGTGGAATAGAAAGGCGGGAAAGGTGGGGAAAAGATTGAGAAATCGGATGGTTGCCGTGTCTGTGTAGAAAGAAGTAGACATGGGAGACTTTTCATTTTGTTCTGCACTAAGAAAGATTCTTCTGCCTTGGGATCCTGTTGATCTGTGACCTTACCCCCAACCCTGTGCTCTCTGAAACATGTGCTGTGTCCACTCAGGGTTAAATGGATTAAGGGCGGTGCAAGATGTGCTTTGTTAAACAGATGCTTGAAGGCAGCATGCTCGTTAAGAGTCATCACCAATCCCTAATCTCAAGTAATCAGGGACACAAACACTGCGGAAGGCCGCAGGGTCCTCTGCCTAGGAAAACCAGACACCTTTGTTCACTTGTTTATCTGCTGACCTTCCCTCCACTATTGTCCCATGACCCTGCCAAATCCCCCTCTGTGAGAAACACCCAAGAATTATCAATAAAAAAAAAAAAAAAAAAAAAAAAAAAAAAAAAAAAAAAAAAAAAAAAAAAAAAAAAAAAAAAAAAAAAAAAAGCAGATACGAagtaggctttccatgtaacttaacaccatttttcattttctttctttttttttttttttttttaaatagaaatagggtcttcctgtgtagcccaggctggcctcgaactcctgggctcaagtgatcctcccgcctcggactctcaaagtgctgggattacaggcatgagccactgagcctgatctatttttttcttatctttttttttaaattgatattttagaatagtagactttcacagcagtgcctcctgcactgataatacagtacaccatttctgcatgtaaatatttaaaataagtcagaacgaacgttaaggcatataaagttactttaagtactaacactattattattattattatttttttgagacagagtttcgttcttgttgcccaggctggagtgcaatggcgcgatctcggctcacctcaacctccgcctcccaggttcaagccattctcctgcctcagcctcgcgagtagctgggattacaggcatgcgccaccatgcccagctaattttgtatttttagcagagttggggtttctccatgttggtcaggctggtctcaaactcctgacctcaggtgatcctcccacctcggcctcccaaagtgctgggattataggcgtgagccaccgcgcccggcctctaacactattttcaataagattatgtgtaagcatggtggtgtgtgcctgtagtcccagctacttgggacactgaggtgggaggattgcttgacccaggaggcagaggttgcagtgagctgagattgtgccactgcactccagcttgggcaacagagtgagactctgtctcaaaaataaaaaaaagaaatagttgtgtttttgaacctatgtggtctctgatttttgttagtggtcttgataggaaaataagtccctaatcctagtattgcactgaatgatgacaagccccagggtttaatctggaaggatctttctattgtgttaaatttctaatagcgtcttttccccctggtttgtctcttctcttgaagttttaacgtaaataactgctgtggtagcctttgaacaggctaactctctgtagggctgagattttgatcacattttaaataagcaccttctctctctttaaagatagactatactgaaatatgtaagatacgcgcattaaatctaatagctctccacatggtcaaatgttcactttacttttaatttgcgattgcagGTAACATTCAGATATCCTGGATTGATGACACATCAGCATTTGTTTCCCTTAGCCAGCCCGAGCAAGTAAAGATTGGTAAGTGTTTTGGATTTCTGTTTTGTGTATTAAATACAGTGAGAGGTTGCCCTCCCACTGCCCCCTTAATTTAAAAGCTATGGGCTCAGTCAGCTTACACTCATTGCTGCCTCGT...
