# Supplementary figures and images for: Optical Genomic Mapping and Next-Generation Sequencing Identified Retrotransposon Insertion and Missense Variant Disrupting PARN Gene in Dyskeratosis Congenita
Source: Hum Mutat. 2025 Aug 22;2025:9290736. doi: 10.1155/humu/9290736 (PMC12396913; doi:10.1155/humu/9290736)

**a**

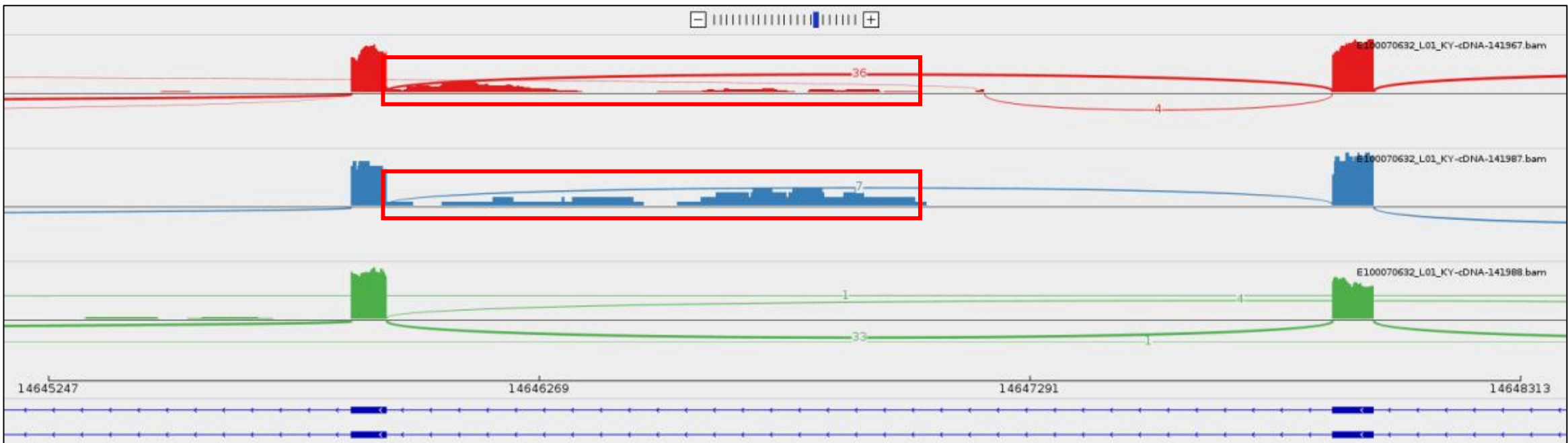

**b**

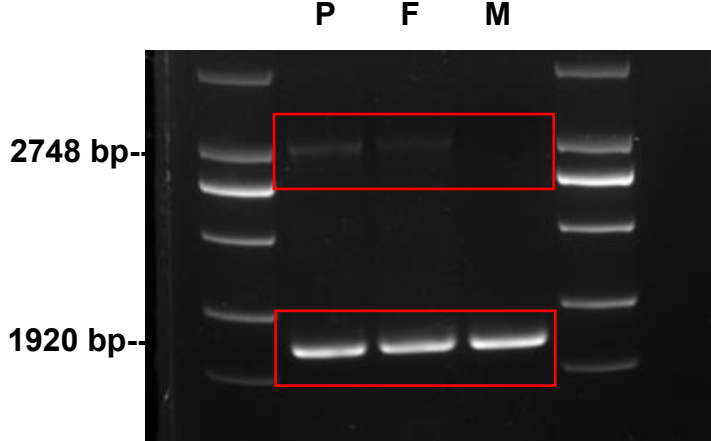

**c**

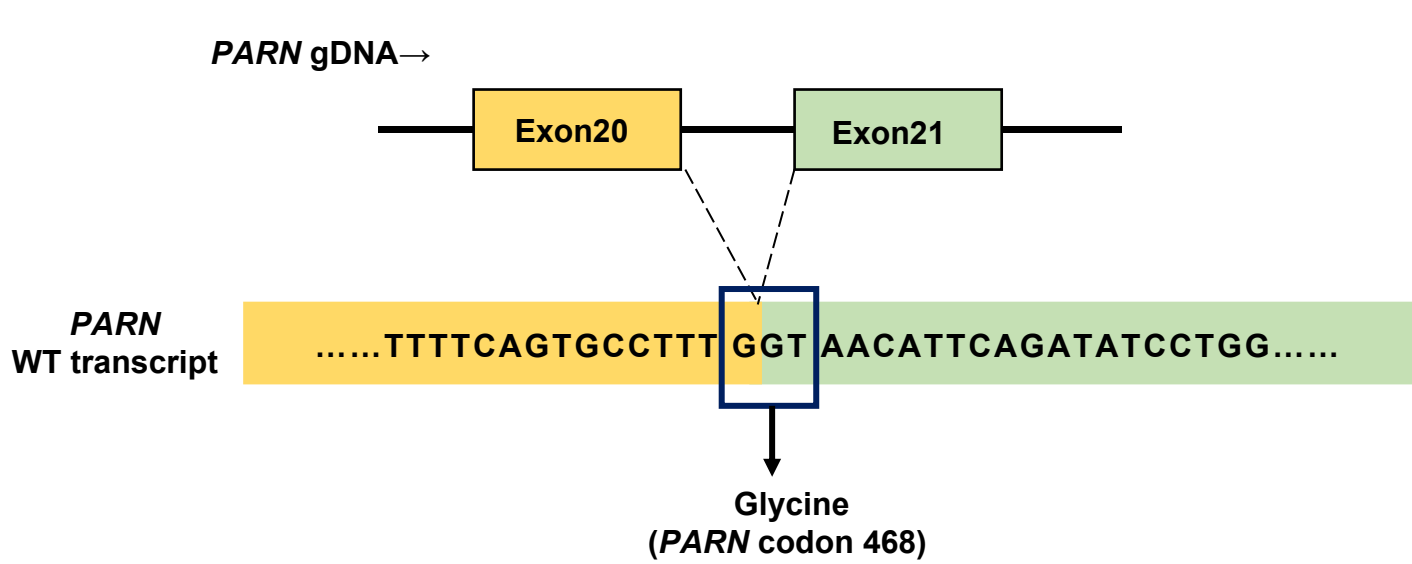

**d**

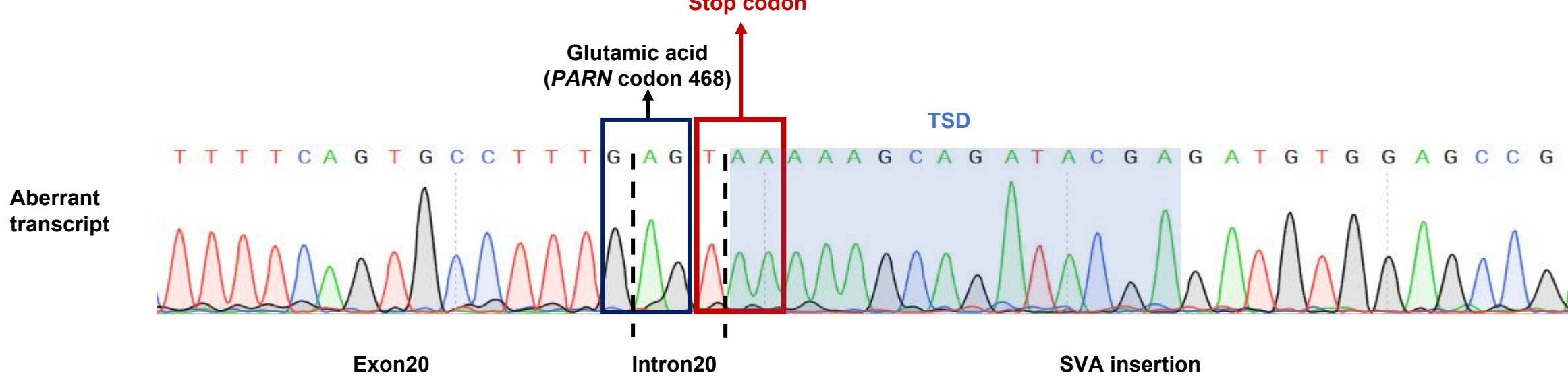

**e**

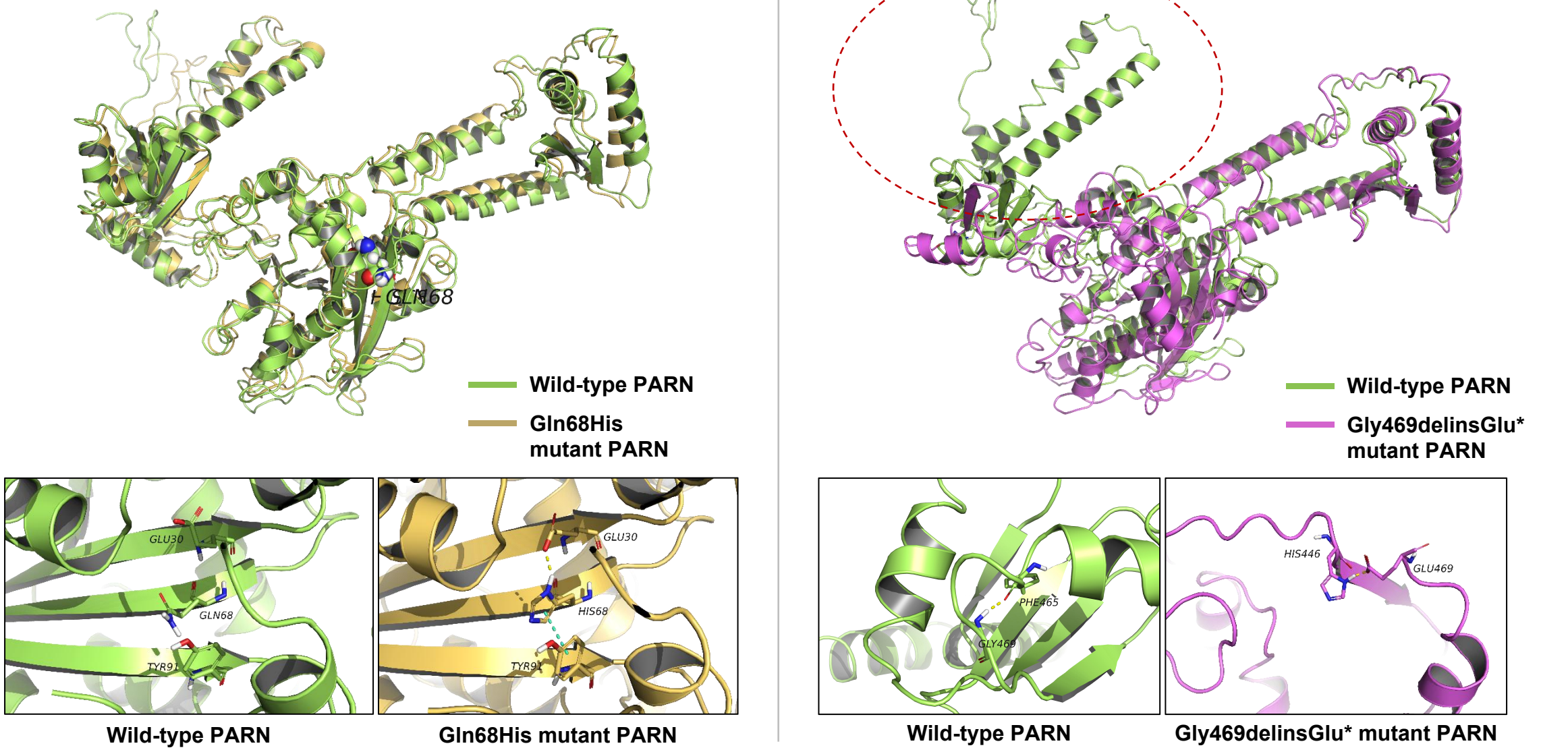

**f**

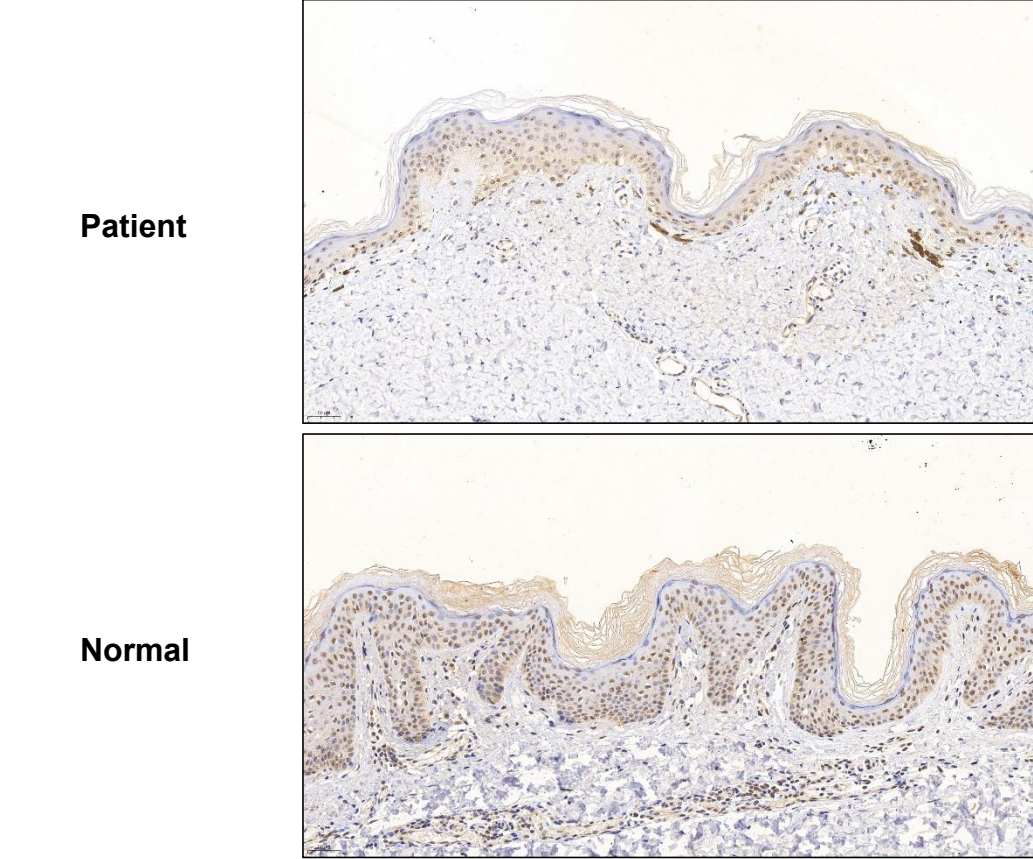

**g**

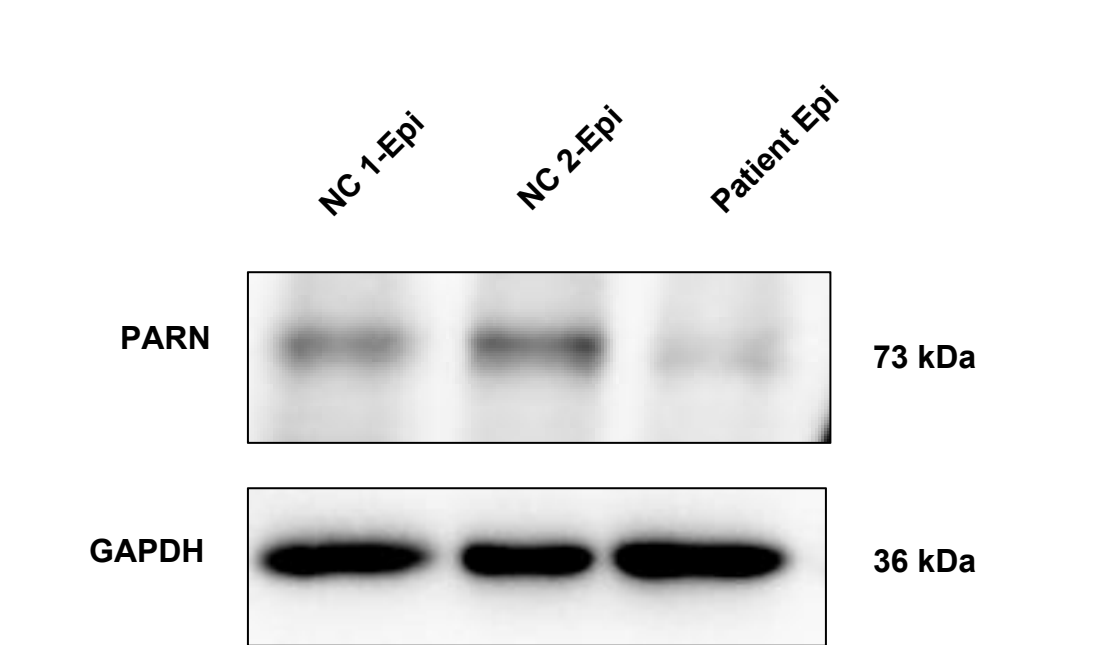

Supplement: Supporting Information 3 — Figure S1: Genetic analysis at the RNA and protein levels, including intron retention, aberrant transcripts, protein structural changes, and reduced PARN expression. [file 9290736.f3.pdf]

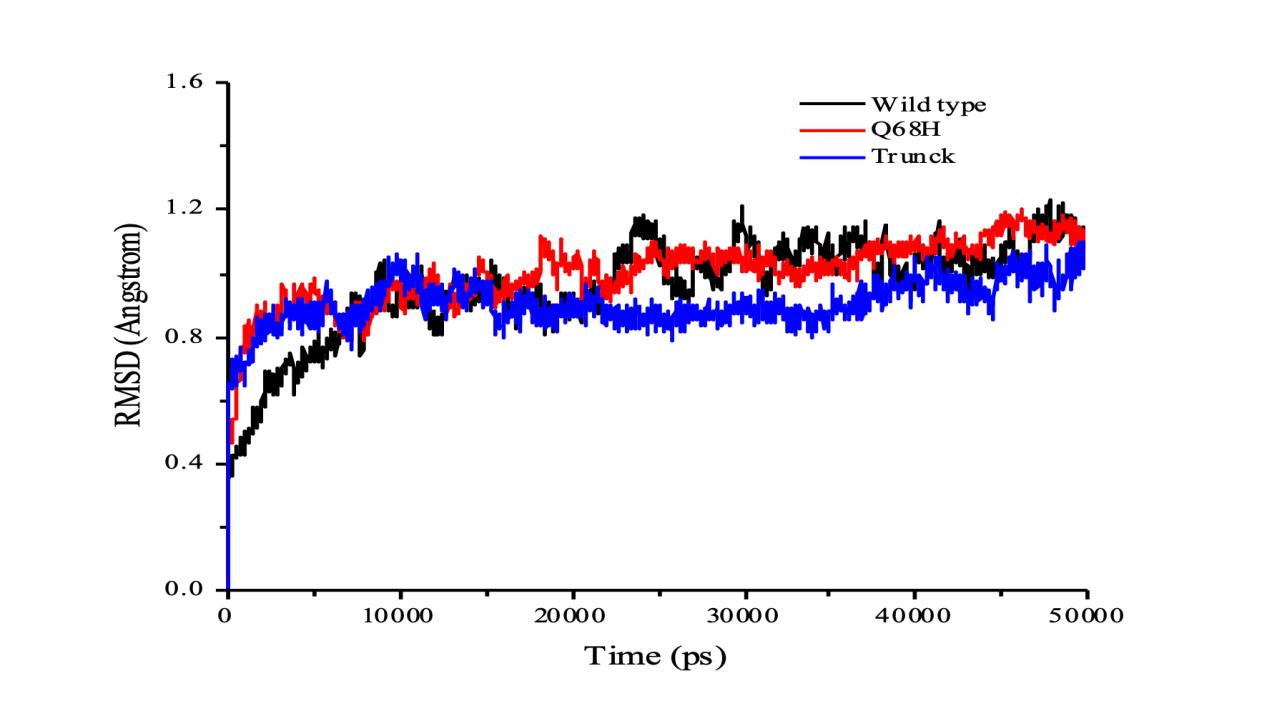

Supplement: Supporting Information 5 — Figure S3: Molecular dynamics simulation results comparing the stability of wild-type and mutant PARN proteins. [file 9290736.f5.jpg]

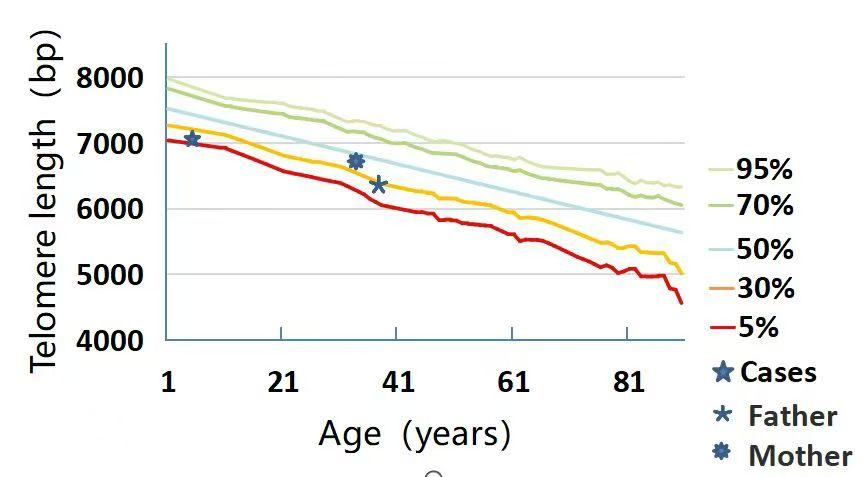

Supplement: Supporting Information 6 — Figure S4: Telomere length analysis, showing reduced telomere length in the proband compared to age-matched controls. [file 9290736.f6.jpg]
