# Supplementary material for: Optical Genomic Mapping and Next-Generation Sequencing Identified Retrotransposon Insertion and Missense Variant Disrupting PARN Gene in Dyskeratosis Congenita
Source: Hum Mutat. 2025 Aug 22;2025:9290736. doi: 10.1155/humu/9290736 (PMC12396913; doi:10.1155/humu/9290736)
Supplement: Supporting Information 4 — Figure S2: The full inserted sequence of the SVA retrotransposon within the PARN gene, including annotated splicing and TSD regions. [file 9290736.f4.pdf]

Intron 20

cttctctttcctttttttaccaggtcatgtgattaggttcgtttgagcagtctcctgataagtgtttatgaagaccattctgattgtaaacccacagcaatgcctcttattttaacaaacgaagcattt
aagctattctctttggaaattcttggataaagtgaaggccacaaaacctaaatgtccagtagtaagaaagtttagtaaatgtttatgtatcttc

c.1450+727

tagtatgtagccatttaaaaaatatttgaggagtttattaaaagaagttaaagtgcttgtattttaacgtttagagtAAAAAGCAGATACGA →

Inserted sequence

GATGTGGAGCCGAAGCTGGACTGTACTGCTGCCATCTCGGCTCACTGCAACCTCCCCGCCTGATTCTCCTGCCTCAGCCTGCCAAGTGCCTGCGAT
TGCAGGCACGCGCCACCACGCCTGACTGGTTTTGGTGGAGACGGGGTTTCGCTGTGTTGGCCGGGCCGGTCTCCAGCCCCTAACCGCGAGTGAT
CCGCCAGCCTTGGCCTCCCGAGGTGCCGGGATTGCAGACGGAGTCTCGTTCACTCAGTGCTCAATGGTGCCCAGGCTGGAGTGCAGTGGCGTGAT
CTCGGCTCACTACAACCTACACCTCCCAGCCGCCTGCCTTGGCCTCCCAAAGTGCCGAGATTGCAGCCTCTGCCCGGCCGCCACCCCGTCTGGGA
AGTGAGGAGTGTCTCTGCCTGGCCGCCCATCGTCTGGGATGTGAGGAGCCCCTCTGCCTGGCTGCCCAGTCTGGAAAGTGAGGAGCGTCTCCGC
CCGGCCGCCATCCCATCTAGGAAGTGAGGAGCGCCTCTTCCCAGCCGCCATCACATCTAGGAAGTGAGGAGCGTCTCTGCCCGGCCGCCCATCGT
CTGAGATGTGGGGAGCGCCTCTGCCCCACCGCCCCATCTGGGATGTGAGGAGCGCCTCTGCCCGGCCGAGACCCCGTCTGGGAGGTGAGGAGC
GTCTCTGCCCGGCCGCCCCGTCTGAGAAGTGAGGAGACCCTCTGCCTGGCAACCACCCCGTCTGAGAAGTGAGGAGCCCCTCCGCCCGGCAGCC
GCCCCGTCTGAGAAGTGAGGAGCCTCTCCGCCCGGCAGCCACCCCATCTGGGAA
GTGAGGAG……..TCTGCTGACCTTCCCTCCACTATTGTCCCATGACCCTGCCAAATCCCCCTCTGTGAGAAACACCCAAGAATTATCAATAAAAAAAA
AAAAAAAAAAAAAAAAAAAAAAAAAAAAAAAAAAAAAAAAAAAAAAAAAAAAAAAAAAAA

AAAAAGCAGATACGA →

Intron 20

agtaggctttccatgtaacttaacaccatttttcattttctttctttttttttttttttttttaaatagaaatagggtcttcctgtgtagcccaggctggcctcgaactcctgggctcaagtgatcctcccg
cctcggactctcaaagtgctgggattacaggcatgagccactgagcctgatctatttttttcttatcttttttttttaaattgatattttagaatagta
