# Supplementary material for: Optical Genomic Mapping and Next-Generation Sequencing Identified Retrotransposon Insertion and Missense Variant Disrupting PARN Gene in Dyskeratosis Congenita
Source: Hum Mutat. 2025 Aug 22;2025:9290736. doi: 10.1155/humu/9290736 (PMC12396913; doi:10.1155/humu/9290736)
Supplement: Supporting Information 7 — Table S1: Lists of all primers used in long-range PCR and RNA validation experiments. [file 9290736.f7.docx]

**SUPPLEMENTARY TABLE**

**Table S1. The forward and reverse primer**

| **Primer Name** | **Sequence (5ʹ - 3ʹ)** |
| --- | --- |
| 1-F | ACGAGGCAGCAATGAGTGTAAGC |
| 1-R | CCATGGCAGAATCCCACCAATCC |
| 2-F | GGATACACTCGCTTCCCTGG |
| 2-R | CCATGGCAGAATCCCACCAATCC |
| 3-F | TGCAGTGCTCACGAGGCAGCAATGAGTGTAAGC |
| 3-R | TAGAACACCTCCATGGCAGAATCCCACCAATCC |
| 4-F | GGATACACTCGCTTCCCTGG |
| 4-R | CCAAGGCAACAGTTCCACAC |
